# IL-16 and MIF: messengers beyond neutrophil cell death

**DOI:** 10.1038/cddis.2015.388

**Published:** 2016-01-14

**Authors:** S Roth, W Solbach, T Laskay

**Affiliations:** 1 Clinic for Infectiology and Microbiology, University of Lübeck, Ratzeburger Allee 160, Lübeck 23562, Germany; 2 Institute for Medical Microbiology and Hygiene, University of Lübeck, Ratzeburger Allee 160, Lübeck 23562, Germany

Most cytokines are synthesized *de novo* upon appropriate stimulation, packed into vesicles and secreted from the producing cell. However, cells can also contain preformed cytokines, which means that these cytokines are synthesized prior to activation and stored inside the cell. Harboring of preformed effector molecules is essential for cells of low transcription and translation activity, as seen in neutrophils. Being part of the first line of defense against invading pathogens, neutrophils are rapidly recruited from the circulation into the infected tissue, where they immediately execute a multitude of antimicrobial effector functions.^1^ Several antimicrobial effector substances are stored inside neutrophils as preformed molecules. This allows a fast action, as these preformed substances can act on demand, without the need of time-consuming *de novo* synthesis. The presence of preformed cytokines in neutrophils emphasizes that these cells are not solely fast-acting effector cells but they can also regulate very rapidly the inflammatory and immune responses.^2^

Recently, we reported the storage of IL-16 and MIF in primary human neutrophils in the new journal *Cell Death Discovery*.^3^ Analysis of neutrophil granule fractions and confocal immune fluorescent microscopy revealed that IL-16 and MIF are stored in the cytosol rather than in neutrophil granules.^3^ Notably, IL-16 is synthesized as a biologically inactive precursor, pre-IL-16 (formerly also termed pro-IL-16), which is processed in a caspase-3-dependent manner. The C-terminal fragment IL-16C is the biologically active, secreted pro-inflammatory cytokine.^4^ Intracellular proteolytic activation by cytosolic proteases has also been described for other pro-inflammatory cytokines such as IL-18, IL-1β and IL-33.^5, 6^

Neutrophils are short-lived cells. After undergoing apoptosis they are cleared from the circulation in liver, spleen and bone marrow. Although their life span is extended at sites of infection/inflammation, large numbers of neutrophils undergo apoptosis after executing their effector functions. Apoptotic cells and blebs maintain their membrane integrity and are cleared by phagocytes in a process called efferocytosis. Since apoptotic cells/blebs are surrounded by an intact cell membrane, this process prevents the leakage/release of potentially harmful cellular content of neutrophils into the surrounding tissue. In case of insufficient or impaired efferocytosis, however, apoptotic neutrophils undergo secondary necrosis, resulting in the leakage of cell content through the disintegrated plasma membrane. Therefore, harboring preformed biologically active molecules not only favors rapid effector functions, but also bears the risk of uncontrolled release during primary necrosis or secondary necrosis. This passive release may lead to tissue damage, but is also believed to contribute to the development of autoimmunity to/against neutrophil constituents.^7^

The secretion mechanism of IL-16C and MIF from neutrophils, but also other cells, remains largely elusive. As both cytokines lack the signal peptide to be transferred to the ER, they are thought to be released by unconventional secretion mechanisms, as known for IL-1 and FGF2.^8, 9^ Previous studies hypothesized that IL-16 and MIF could be released actively from the producing cells.^10, 11^ However, our data indicate that in neutrophils both cytokines are released passively after the cells undergo secondary necrosis^3^ (Figure 1). After the screening of a large panel of substances, among others, TLR ligands, pro-inflammatory cytokines, lipid mediators, glucocorticoids, and the bacteria *Escherichia coli* and *Staphylococcus aureus*, no release of IL-16C and MIF was observed from viable or apoptotic neutrophils.^3^ Instead, release of IL-16C and MIF correlated strongly with the secondary necrosis of neutrophils.^3^

As neutrophils release IL-16C and MIF only after undergoing secondary necrosis, it is tempting to speculate that IL-16C and MIF function as danger signals in case of insufficient clearance of apoptotic neutrophils. At sites of acute infection or inflammation, efferocytosis may not be sufficient to clear the large numbers of apoptotic neutrophils. Consequently, apoptotic neutrophils undergo secondary necrosis. Although the release of IL-16C and MIF from secondary necrotic neutrophils may be required to maintain and modulate the local defense and inflammatory functions, we have to keep in mind that they may also be detrimental in conditions of autoimmunity.

Neutrophils, although known for a long time and studied extensively, are more complex and sophisticated in their functions than was imaginable one or two decades ago. By the release of neutrophil extracellular traps, they can fulfill antimicrobial effector functions even beyond their death.^12^ The study published in *Cell Death Discovery*^3^ extends this view by demonstrating that neutrophils can fulfill even their regulatory functions beyond their life span.

## Figures and Tables

**Figure 1:**
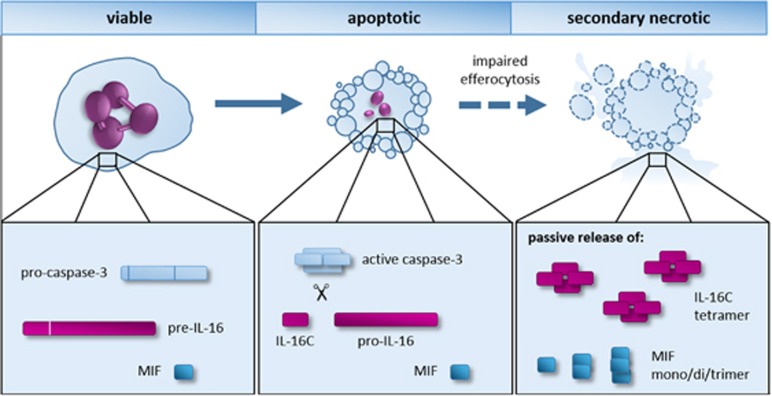
Storage, processing and release of IL-16 and MIF in human neutrophils. MIF and IL-16 are preformed cytokines stored in the cytosol of human neutrophils. During apoptosis, the precursor of IL-16 (pre-IL-16) is processed into the biologically active IL-16C in a caspase-3-dependent manner. Under normal conditions, apoptotic cells are cleared by phagocytes. If the clearance of apoptotic cells is impaired, as it occurs in infections and autoimmunity, apoptotic neutrophils undergo secondary necrosis. IL-16C and MIF are then passively released from secondary necrotic neutrophils
